# Acceptance of Cultured Meat in Germany—Application of an Extended Theory of Planned Behaviour

**DOI:** 10.3390/foods11030424

**Published:** 2022-01-31

**Authors:** Jacqueline Dupont, Tess Harms, Florian Fiebelkorn

**Affiliations:** Didactics of Biology, School of Biology and Chemistry, Osnabrück University, Barbarastr. 11, 49076 Osnabrück, Germany; t.harms29@web.de (T.H.); florian.fiebelkorn@uni-osnabrueck.de (F.F.)

**Keywords:** cultured meat, attitudes, perceived behavioural control, subjective norms, food technology neophobia, path model

## Abstract

This study examines the willingness to consume a cultured meat burger in Germany. Based on the theory of planned behaviour (TPB), we assessed attitudes, perceived behavioural control, and subjective norms via an online questionnaire. Attitudes were operationalized in this research as general attitudes towards cultured meat and specific attitudes towards a cultured meat burger. Furthermore, the TPB was extended with nutritional-psychological variables including food (technology) neophobia, food disgust, sensation seeking, and green consumption values. In total, 58.4% of the participants reported being willing to consume a cultured meat burger. Using a path model, the extended TPB accounted for 77.8% of the variance in willingness to consume a cultured meat burger. All components of the TPB were significant predictors except general attitudes. The influence of general attitudes was completely mediated by specific attitudes. All nutritional-psychological variables influenced general attitudes. Food technology neophobia was the strongest negative, and green consumption values were the strongest positive predictor of general attitudes. Marketing strategies should therefore target the attitudes of consumers by encouraging the natural perception of cultured meat, using a less technological product name, enabling transparency about the production, and creating a dialogue about both the fears and the environmental benefits of the new technology.

## 1. Introduction

The world’s population is projected to increase to 9.7 billion by 2050 [1]. This substantial growth will cause an increased demand for food. As a result, global demand for animal food products will rise as well, particularly in developing countries [2]. However, high meat consumption increases the risk of cardiovascular diseases and cancer [3,4,5,6]. Moreover, the livestock industry has various negative effects on the environment. The livestock industry is a major cause of climate change, accounting for between 14.5% [7] and 51% [8] of global greenhouse gas emissions. In addition to its impact on climate change, industrial livestock production involves high land usage. Of the global arable land, 71% is used for animal feed production alone [9]. Moreover, the treatment of livestock as industrial goods raises many ethical concerns [10]. For all these reasons, expanding livestock farming is not a viable solution to meet the anticipated rise in global demand for meat.

To counteract the ‘diet–environment–health trilemma’ [11] for current and future generations, it is imperative to introduce sustainable and ethically justifiable alternatives to conventional meat production and consumption. Over the last few years, sustainable meat alternatives, including plant-based protein sources such as tofu, seitan, tempeh, and mycoproteins [10,12], as well as insects [13] have gained the attention of researchers and the media. The production of cultured meat offers another sustainable alternative to conventional meat production. Note that although there are different names for cultured meat such as cultivated meat, clean meat, synthetic meat, or in-vitro meat [14], it will be referred to as ‘cultured meat’ throughout this study. Grown from animal stem cells outside the animal’s body and without killing the animal, cultured muscle tissue can be used in various meat products [15]. For instance, the first cultured meat product to be presented to the public was a cultured beef burger patty in 2013 [16].

Although cultured meat is not yet on the European market due to technical obstacles and legal barriers, it was first approved in Singapore in 2020 in the form of chicken nuggets [17]. In addition, several companies around the globe are preparing to enter the market, for example, the Netherlands, the United States, and Israel [15,18,19]. In Germany, the Wiesenhof company is already collaborating with Israeli start-up SuperMeat to manufacture cultured meat [20]. Moreover, it is highly likely that cultured meat will be covered by Regulation (EU) 2015/2283 on novel foods [21]. The German government expects that cultured meat will be marketable in 10 to 20 years [19].

Large-scale production of cultured meat faces technical challenges, such as meeting nutritional requirements [22], and ethical issues, such as finding alternatives to foetal bovine serum as an important part of the culture medium [15,16]. Moreover, the energy consumption necessary to produce cultured meat is still higher than conventional meat products such as beef, pork, mutton, and poultry [23,24]. However, using sustainable energy sources could possibly result in cultured meat having a lower footprint than pork and beef and a similar carbon footprint to chicken meat [25]. In contrast, if conventional energy sources were still used in the production of cultured meat, it would only have a lower carbon footprint than beef but not chicken or pork [25]. Nevertheless, cultured meat may have a smaller overall impact on the environment than conventional meat due to the reduced use of land and water [15,24]. The consumption of cultured meat is also assumed to be healthier than conventional meat due to its lower fat content and the opportunity to add nutrients to cultured meat products [15]. According to a report by A.T. Kearny [26], cultured meat has a high commercial potential for market share, expected growth, and price competitiveness in the next decade. However, despite the anticipated commercial launch of cultured meat within the next few years, as well as its positive effects on the environment, health, and economics, its integration in the market can only succeed if it is accepted by consumers.

### Aims of the Study

Therefore, this study analyses the willingness to consume a cultured meat burger based on the theory of planned behaviour (TPB). Based on the TPB, we aim to determine whether attitudes, subjective norms, and perceived behavioural control influence the intention to consume a cultured meat burger. Onwezen et al. [27] have already shown that the TPB is suitable for explaining the willingness to consume a cultured meat burger. Moreover, they were able to show that the predictive power could be increased by expanding the TPB to include affective factors, such as disgust [27]. Consequently, to predict the intention to consume a cultured meat burger in more detail, this study tested an extended model of the TPB including various sociodemographic and nutritional-psychological variables. Based on the findings, conclusions can be drawn for marketing strategies and information campaigns.

In more detail, we analysed the extent to which the TPB variables ‘attitudes’, ‘subjective norms’, and ‘perceived behavioural control’ influence the willingness to consume a cultured meat burger. In particular, the influence of subjective norms on the acceptance of alternative protein sources has been poorly studied so far [28]. In addition, we assessed the extent to which the nutritional-psychological variables ‘food disgust’, ‘food neophobia’, ‘food technology neophobia’, ‘sensation seeking’, and ‘green consumption values’, as well as the sociodemographic variables ‘gender’, ‘age’, ‘education’, ‘meat consumption’ and ‘meat reduction’ may be relevant to the willingness to consume a cultured meat burger. Sensation seeking and green consumption values have rarely been studied regarding the acceptance of cultured meat. In addition, mediation effects between individual variables will also be considered in this study. In the following section, all variables examined in this study are described and the current state of research is presented.

## 2. Theoretical Framework: An Extended TPB

In environmental and nutritional psychology, the TPB is a frequently used model in the study of sustainable and environmentally friendly behaviour [29,30]. For example, the TPB has already been successfully applied to explain various forms of sustainable behaviour, including waste reduction, energy conservation, and sustainable nutrition [31,32,33,34,35,36,37,38,39,40,41]. In addition, the TPB has also been used in the study of the acceptance and willingness to consume novel foods. In this context, the TPB has been successfully applied in various studies to predict acceptance, willingness to consume, and willingness to purchase insect-based foods [27,42,43,44,45,46,47]. Furthermore, Onwezen et al. [27] were able to show that the TPB is also suitable to explain the acceptance of fish, seaweed, pulses, and cultured meat.

According to Ajzen [31], the behavioural intention—in this study the willingness to consume a cultured meat burger—to execute a certain behaviour is the immediate antecedent of the behaviour. The intention is an aggregate of motivating factors and efforts an individual is willing (or not willing) to invest to perform a certain behaviour [31]. Moreover, the intention is driven by three determinants, namely attitudes, perceived behavioural control and subjective norms [32,46]. The TPB postulates that the more positive a person’s attitude, and subjective norms are towards a particular behaviour, and the greater the perceived behavioural control, the stronger the person’s intention to perform the behaviour in question [32].

**Figure 1:**
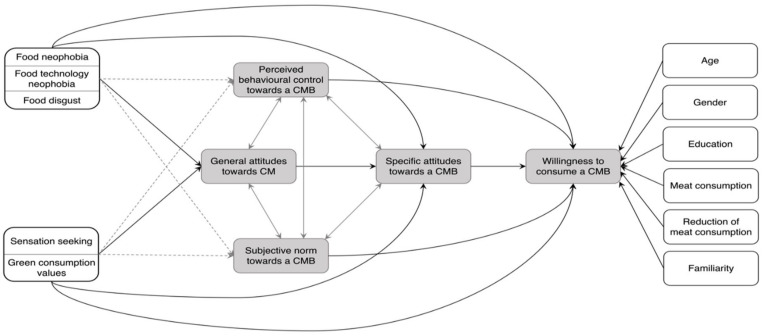
Theoretical framework of the study based on the Theory of Planned Behaviour (grey) and its extended variables (white). Black arrows indicate hypothesised relationships. The grey dashed lines represent the anticipated exploratory relationships in the model. Grey double arrows represent correlations between the variables. Note: CM = Cultured meat, CMB = Cultured meat burger.

In addition to the main components of TPB, Ajzen [31,32], Menozzi et al. [46], and Onwezen et al. [27] proposed that the prediction of an intention can be increased by adding other variables, also called background factors [32]. According to Ajzen [32], the effects of the background factors are more likely mediated by attitudes than by subjective norms and perceived behavioural control. Nevertheless, the influences of the background factors on subjective norms and perceived behavioural control were also analysed in this study (Figure 1).

### 2.1. Variables of the TPB

#### 2.1.1. Attitudes

Attitudes can be defined as affective, cognitive, or behavioural evaluations of an attitude object. These ‘attitude objects’ can consist of either concrete items, such as a cultured meat burger, or abstract entities [48], such as activities and behaviours. Thus, attitudes evaluate an attitude object as favourable or unfavourable [31]. This study surveyed general attitudes towards cultured meat, as well as attitudes towards a specific product, a cultured meat burger. Consumer attitudes are important predictors of acceptance that vary significantly as they are influenced by several factors [49]. According to Dupont and Fiebelkorn [50], attitudes towards a cultured meat burger have been shown to be the most important factor in predicting the willingness of children and young people to consume a cultured meat burger. Similarly, attitudes toward an insect-derived burger were identified as the most important factor in predicting willingness to consume an insect burger. Furthermore, Dupont and Fiebelkorn [50] were able to show that general attitudes towards the two novel foods also have a positive influence—albeit a smaller influence than attitudes towards the burger—on the willingness to consume the two burgers. Consistent with these results, Kroesen and Chorus [51] assume there is a stronger relationship between behaviour and attitudes when the two constructs show high correspondence with their target. In the present study, we used two different goals, with attitudes toward the cultured meat burger compared to attitudes toward cultured meat showing a higher correspondence with our measured behavioural intention—the willingness to consume a cultured meat burger. Therefore, it is expected that attitudes toward a specific product, namely a cultured meat burger, have a stronger influence on the willingness to consume the burger than attitudes toward the food without reference to a specific product category (cultured meat). In addition, following the composite model of attitude–behaviour relationships, a distinction can be made between two different levels of attitudes [52]. According to this model, the impact of attitudes toward the goal of the behaviour is moderated by the attitudes towards an explicit behaviour [52]. In the present study, although both attitude variables can be understood as goal attitudes, as previously described, the specific attitudes are more closely related to our behavioural intention. Consequently, it could be assumed that the influence of general attitudes toward cultured meat is mediated by specific attitudes toward the cultured meat burger.

Although attitudes had a significant influence on willingness to consume a cultured meat burger in the study by Onwezen et al. [27], they were not the strongest predictor. Bryant et al. [53] were also able to show that various attitudinal dimensions, such as healthiness or appeal, had an impact on the willingness to purchase cultured and plant-based meat in respondents from the United States, China, and India—countries with significant differences in attitudinal dimensions.

#### 2.1.2. Subjective Norms

‘Subjective norms’ describes the perceived social pressure from people who are important in one’s life [31]. Other people’s expectations can affect the formation of behavioural intentions and eventually, the behaviours of a person [54]. In previous subjective norms have often displayed a relatively weak influence on willingness to consume sustainable foods compared to attitudes and perceived behavioural control [32,33,46]. Note, however, that the influence of subjective norms on behavioural intention can be partly moderated by perceived behavioural control [55]. However, in a study by Onwezen et al. [27], subjective norms were the strongest predictor of willingness to consume not only a cultured meat burger but also a fish burger, a seaweed burger, a pulse burger, and an insect burger. In contrast, in a study by Menozzi et al. [46], subjective norms had the weakest influence on willingness to eat an insect-based food product. Moreover, Chang et al. [43], revealed that subjective norms even displayed no influence on willingness to purchase insect-based foods. 

#### 2.1.3. Perceived Behavioural Control

Perceived behavioural control is defined as the perceived degree of ease or complication one experiences when performing a certain behaviour [31,54]. A potential factor influencing perceived behavioural control, for instance, is an individual’s situation-specific confidence in his or her ability to perform a behaviour. The range of resources and opportunities, for instance, temporal or financial, or the lack thereof [31], also affect a person’s perceived behavioural control. According to Onwezen et al. [27], perceived behavioural control has a positive influence on willingness to consume a cultured meat burger. Menozzi et al. [46] also showed that perceived behavioural control has a positive influence on willingness to consume insect-based food products.

### 2.2. Extending the TPB

#### 2.2.1. Sociodemographic Data and Meat Consumption

##### Age, Gender, Education

Previous research reported that males and younger people had more positive attitudes towards cultured meat and were more willing to try it, compared with females and older people [49,56]. Bryant et al. [57] were also able to show in their study that younger German consumers were more willing to buy cultured meat. Gender, on the other hand, did not influence the willingness to buy cultured meat [57]. In addition, in previous studies, respondents with a higher level of education showed a higher willingness to buy and try cultured meat [58,59,60]. In contrast, in a study by Weinrich et al. [61], educational level, age, and gender had no influence on willingness to consume cultured meat among German respondents.

##### Meat Consumption

Previous research proved that high meat consumption leads to increased willingness to try cultured meat [58]. In addition, Bryant et al. [53] showed that Chinese and Indian participants with higher meat consumption were more willing to purchase cultured meat compared to participants with lower meat consumption. Additionally, Dupont and Fiebelkorn [50] demonstrated that German children and adolescents with higher meat consumption were more willing to consume a cultured meat burger.

##### Reduction of Meat Consumption

In a study by Dupont and Fiebelkorn [50], no influence of the intention to reduce one’s meat consumption on the acceptance of trying a cultured meat burger could be shown. In contrast, Wilks et al. [60] were able to show that respondents who did not restrict their meat consumption showed a higher acceptance of cultured meat. Moreover, Wilks and Phillips [62] demonstrated a negative correlation between a vegetarian or vegan lifestyle and the willingness to consume cultured meat.

#### 2.2.2. Nutritional-Psychological Variables

##### Familiarity

Following Bryant et al. [53] respondents from three countries (the United States, China, and India) who had higher familiarity with cultured meat showed greater willingness to purchase it. Furthermore, in other studies, a greater familiarity with cultured meat has been associated with its increased acceptance [62,63], though this finding was not statistically tested.

##### Food Disgust

‘Food disgust’ is defined as a person’s disgust sensitivity to certain food-related cues based on stimuli such as contamination or decay [64]. In a recent paper, Siegrist and Hartmann [65] were able to demonstrate a negative influence of food disgust on the acceptance of cultured meat. In addition, an influence of food disgust on the disgust triggered by cultured meat could be proven [65]. Bryant et al. [53] also demonstrated an influence of disgust on willingness to purchase cultured meat in American participants. In contrast, in research by Dupont and Fiebelkorn [50] with German children and adolescents, no influence of food disgust on the willingness to eat a cultured meat burger could be demonstrated.

##### Food Neophobia

‘Food neophobia’ is defined as the aversion to novel foods [66]. Wilks et al. [60] have shown that attitudes towards cultured meat and willingness to eat it are predicted by food neophobia. That is, individuals with higher food neophobia have more negative attitudes and a lower willingness to eat cultured meat. Siegrist and Hartmann [65] also demonstrated a negative effect of food neophobia on the acceptance of cultured meat as well as on the disgust caused by cultured meat. In addition, Bryant et al. [53] were able to demonstrate for the Chinese and American participants in their study that food neophobia reduces the willingness to purchase cultured meat. For Indian participants, food neophobia did not show any influence on the willingness to purchase cultured meat [53]. According to Dupont and Fiebelkorn [50], food neophobia negatively influenced the willingness to eat a cultured meat burger and an insect burger by German children and adolescents.

##### Food Technology Neophobia

Food technology neophobia is described as the reluctance to eat food produced using novel technologies [67]. Limited research has appeared on the impact of food technology neophobia on the willingness to consume cultured meat. However, Gómez-Luciano et al. [68] showed that fear of novel food technologies had no effect on willingness to purchase cultured meat among participants from the United Kingdom, Spain, Brazil, and the Dominican Republic. In contrast, Bryant et al. and Siegrist et al. [69,70] demonstrated that the more information about the production of cultured meat was framed technically, the less positive its evaluation by consumers and the lower the intention to try, buy or eat cultured meat as a replacement for conventional meat. In addition, a negative influence of food technology neophobia has been shown for the willingness to consume other novel foods, such as insect-based foods [71,72].

##### Sensation Seeking

‘Sensation seeking’ describes the need for new and intense adventures as well as the closely related willingness to take risks [73]. Although no comparable data relates the influence of sensation seeking to the willingness to eat cultured meat, findings concerning the consumption of insect-based food products indicate a positive influence of sensation seeking on the willingness to consume insect-based products [74,75].

##### Green Consumption Values

‘Green consumption values’ depict the impact of an individual’s personal ecological and environmental values on consumption and purchase behaviour [76]. It has been found that strong environmental concern contributes to sustainable consumption behaviour [77]. In addition, previous studies have shown that many consumers are cognizant of the benefits of cultured meat for the environment and animal welfare [56,78,79]. Circus and Robison [80] were also able to show that environmental friendliness is one of the main reasons for consuming cultured meat. Moreover, it was demonstrated that providing information about the sustainability aspects of cultured meat positively affects consumers’ attitudes towards the product [63].

### 2.3. Research Questions and Hypotheses

The main aim of this study was to analyse to what degree attitudes, subjective norms, and perceived behavioural control influence the willingness to consume a cultured meat burger. Based on the literature cited above, the following hypotheses were formulated (see also Figure 1):

- H1.1: Specific attitudes towards a cultured meat burger have a positive influence on the willingness to consume a cultured meat burger.
- H1.2: Specific attitudes towards a cultured meat burger act as a mediator between general attitudes towards cultured meat and the willingness to consume a cultured meat burger.
- H1.3: Subjective norms have a positive influence on the intention to consume a cultured meat burger.
- H1.4: Perceived behavioural control has a positive influence on the willingness to consume a cultured meat burger.
- H1.5: Specific attitudes towards a cultured meat burger have the strongest positive influence on the willingness to consume followed by perceived behavioural control and subjective norms.

Additionally, our study analysed the degree to which sociodemographics, food disgust, food neophobia, food technology neophobia, sensation seeking, and green consumption values are predictors of general attitudes towards cultured meat and the willingness to consume a cultured meat burger.

- H2.1: Younger males and better-educated participants will demonstrate a higher willingness to consume a cultured meat burger.
- H2.2: Respondents with high meat consumption or who do not want to reduce their meat consumption will show a higher intention to consume a cultured meat burger.
- H2.3: Higher familiarity with cultured meat leads to a higher willingness to consume a cultured meat burger.
- H2.4: Food disgust, food neophobia, and food technology neophobia have a negative impact on general attitudes towards cultured meat.
- H2.5: The willingness to consume a cultured meat burger is negatively influenced by food disgust, food neophobia, and food technology neophobia.
- H2.6: Sensation seeking and green consumption values will show a positive impact on general attitudes towards cultured meat.
- H2.7: Sensation seeking and green consumption values positively influence the willingness to consume a cultured meat burger.

## 3. Materials and Methods

### 3.1. Study Design and Sample

An online questionnaire was constructed via the platform SoSci Survey and launched in cooperation with the research panel provider Consumerfieldwork GmbH [81] on 19 June 2019. Selection criteria included a sample size of at least 500 respondents and a minimum age of 18 years. Participants who completed the questionnaire were reimbursed €2. A total of 512 cases were collected. However, 15 participants were excluded before the analysis due to missing values. Accordingly, only 497 cases were included in the analysis. The sample consisted of 249 (50.1%) male and 248 (49.9%) female participants. The gender distribution of the sample approximately matched the overall population of Germany, which is 49.3% male and 50.7% female [82]. Age varied from 18 to 86 years with a mean age of 49.9 years (*SD* = 16.23). The mean age was slightly higher than that of the German population at 44.4 years [83]. For education, a perceptible tendency towards higher education was seen compared to the German population [82]. Namely, more than half of the sample reported having acquired university entrance qualification (55.4%), 44.1% had completed secondary school and 0.4% indicated having no school-leaving qualification. Ten (2%) participants stated they were vegan, while 27 (5.4%) and 136 (27.4%) identified themselves as vegetarian and flexitarian, respectively. Compared to the distribution of diet types within the German population (Flexitarians: 29.1–15%; Vegetarians: 6–4.4%; Vegans: 3.2–2%) [84,85], the proportions of flexitarians, vegetarians, and vegans were somewhat higher in this study.

### 3.2. Measuring Instrument and Variables

At the beginning of the online survey, a short explanation of the meaning of cultured meat was presented: ‘It is now possible to produce meat in the laboratory. Scientists also call this meat “cultured meat”. The production of “cultured meat” requires little space and the suffering of the animals is avoided. Therefore, its consumption has advantages for the environment and in terms of sustainable nutrition.’ This description was formulated based on Verbeke [72] and Bryant and Dillard [69]. The remainder of the questionnaire was divided into five segments that solicited information from respondents regarding their (1) eating habits (diet, meat consumption, intention to reduce meat consumption), (2) nutritional-psychological variables (food disgust, food neophobia, food technology neophobia, sensation seeking, and green consumption values), (3) knowledge of cultured meat as a foodstuff (familiarity, previous consumption), (4) constructs of the TPB (intention to consume a cultured meat burger, attitudes towards cultured meat, specific attitudes towards a cultured meat burger, perceived behavioural control, and subjective norm), and (5) sociodemographic data, respectively. The resulting variables of the aforesaid constructs were based on the German versions of the scales already used by Lammers et al. [74], Kusch and Fiebelkorn [76], and Dupont and Fiebelkorn [50]. The response formats for the scales were also adopted from the studies mentioned above to ensure better comparability. Inverse items were recoded accordingly. The scale examining green consumption values contained a bad quality item (‘Please now click on “do not agree at all” on the far left to prove that you read the texts.’) to check whether participants had read the statements properly. In total, 17 respondents were excluded from the analysis because they failed this bad quality item. These cases were not considered in the sample size. Table 1 summarises the descriptive statistics of the measures. The original questionnaire can be obtained upon request from the corresponding author.

#### 3.2.1. Theory of Planned Behaviour

##### Willingness to Consume a Cultured Meat Burger

In the current study, the ‘intention to consume a cultured meat burger’ will be used synonymously with the ‘willingness to consume a cultured meat burger’, since it represents the behavioural intention that this study wishes to capture. Following Lammers et al. [74], the construct ‘willingness to consume’ consists of three items assessing the extent to which participants are willing to try and buy a cultured meat burger, as well as how willing they are to use the cultured meat burger as a meat substitute [54]. The burger was chosen as a food product because the first public presentation of cultured meat was a burger patty [86]. Moreover, a burger was chosen because of existing equivalent surveys involving vegetarian, insect, and cultured meat burgers [27,50,59,74,76]. The respondents scored each item on a 7-point bipolar scale (–3 = very unlikely; +3 = very likely). For example, willingness to try was assessed by the question, ‘How likely is it that you would try the cultured meat burger?’ The scale was transformed into a 7-point unipolar scale for analysis (Table 2) and displayed high internal consistency (α = 0.94).

##### Attitudes

In this study, attitudes were measured via two constructs: general attitudes towards cultured meat and specific attitudes towards a cultured meat burger, which were based on a visual presentation of a burger patty made of cultured meat. Items of the general attitude scale have cultured meat as a broad target, not a specific product, while the items of the specific attitude scale have a cultured meat burger as a target. The willingness to consume was also explicitly surveyed for eating a cultured meat burger. Therefore, greater correspondence was seen between both the willingness to consume and the specific attitudes towards a cultured meat burger than with the general attitudes towards cultured meat [87].

##### General Attitudes towards Cultured Meat

General attitudes towards cultured meat were measured based on a scale developed by Ruby et al. [75] to assess attitudes towards insects as food. In this survey, we substituted the term ‘cultured meat’ for ‘insects’ in seven items, while two items were completely reworded due to their insect-specific meaning: ‘It is unconscionable to produce cultured meat’ and ‘No animals have to suffer when cultured meat is produced’. Following Dupont and Fiebelkorn [50], items were scored on a 5-point Likert scale. Answer options ranged from ‘1 = I do not agree’ to ‘5 = I fully agree’, with six of the items reverse coded (Table 3). Due to technical difficulties, the item ‘Eating cultured meat is good for the environment’ was not displayed to the participants in the online questionnaire and was therefore excluded from the scale. The scale had a high internal consistency (α = 0.85).

##### Specific Attitudes towards a Cultured Meat Burger

**Figure 2:**
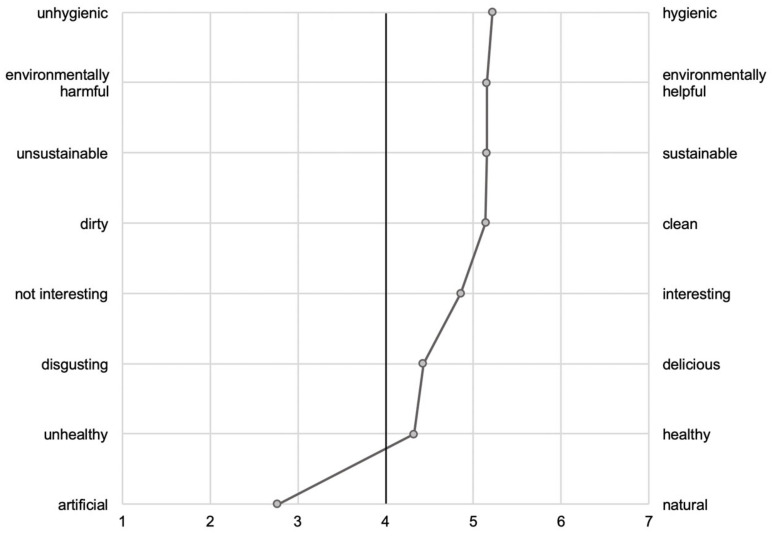
Polarity profile displaying the eight adjective pairs describing the attitudes towards a cultured meat burger. The solid black line at scale point 4 marks the scale centre. The pairs of adjectives represent the scale endpoints (e.g., 1 = unhygienic and 7 = hygienic).

In line with Breckler [48], Hartmann et al. [88], and Bryant et al. [53], attitudes towards a cultured meat burger were measured using a semantic differential. Respondents were shown an image of a cultured meat burger, then their attitudes were assessed using eight items represented by pairs of opposite adjectives verbally anchored to the extreme values and graded on a 7-point bipolar scale (see Table 4 and Figure 2). The smallest value was linked to a negative connotation (e.g., –3 = disgusting) and the highest value to a positive connotation (e.g., +3 = delicious). For the analysis, the bipolar scale values were recoded into unipolar scale values (Table 4). The eight items displayed high internal consistency (α = 0.91).

##### Perceived Behavioural Control

Following De Groot and Steg [89], to assess perceived behavioural control, participants were asked to respond to the question, ‘How easy would it be to integrate the cultured meat burger into your regular diet?’ The extreme categories were anchored verbally (–3 = very difficult; +3 = very easy) and the other categories numerically. Thus, perceived behavioural control was measured with a 7-point bipolar scale, which was transformed into a 7-point unipolar scale for analysis (Table 1).

##### Subjective Norms

To assess subjective norms, the participants should indicate on a 7-point bipolar scale how likely they think that people who are important to them (family and friends) would try, buy, or use the cultured meat burger as a meat substitute. The bipolar scale values (–3 = very unlikely; +3 = very likely) were transformed into unipolar scale values for analysis (Table 1). The variable proved to have high internal consistency (α = 0.93).

#### 3.2.2. Extension of the Theory of Planned Behaviour

##### Sociodemographic Data (Gender, Age, Education)

For sociodemographic data, we assessed age, gender, and education. While gender was coded dichotomously (‘1 = male’; ‘2 = female’), information about education was surveyed in compliance with the standards of acquisition of educational level according to the Statistisches Bundesamt (Destatis) [90]. Thus, the respondents could answer by choosing from the following options: (1) ‘Student’, (2) ‘Without school–leaving qualification’, (3) ‘Lower secondary school’, (4) ‘Higher secondary school’, (5) ‘University entrance qualification’, (6) ‘Abitur’ and (7) ‘Another school degree, namely:’. The respondents’ answers in the free text field could all be assigned to the given answer options.

##### Meat Consumption

Meat consumption was measured by assessing the number of days per week when participants tend to eat meat. The response options included ‘1 = Never’, ‘2 = Once or twice’, ‘3 = Three to four times’, ‘4 = Five to six times’ and ‘5 = Daily’ [73]. In addition, respondents were able to indicate other consumption frequencies using an open text box. These answers were coded to the given options, where possible. As this was not possible in all cases, we coded the data as follows: ‘1 = Never’, ‘2 = Once to three times a month’, ‘3 = Once or twice a week’, ‘4 = Three to four times a week’, ‘5 = Five to six times a week’ and ‘6 = Daily’. Vegetarians and vegans were placed in the group of people who never consume meat.

##### Reduction of Meat Consumption

The intention to reduce one’s consumption of meat, such as beef, pork, and poultry, was measured as a single dichotomous item based on Verbeke [72]. Vegetarians and vegans were classified as participants without meat consumption (‘1 = no meat reduction’; ‘2 = meat reduction’, ‘3 = no meat consumption’) [50,74].

##### Familiarity with Cultured Meat

Respondents were asked about their familiarity with cultured meat. Their response options included ‘1 = No, I’ve never heard of it.’, ‘2 = Yes, I’ve heard of it, but I don’t know what it means.’ and ‘3 = Yes, I’ve heard of it, and I know what it means.’ [72]. In addition, participants were asked to indicate from whom or where they had heard about cultured meat. Respondents could answer by choosing from the following options: ‘Friends/Acquaintances’, ‘Television’, ‘Internet’ and ‘Newspaper’. Moreover, participants were able to provide further sources of information via a free text field. Where possible, these answers were integrated into existing categories, or new response categories such as ‘Radio’ were created (Table 5).

##### Food Neophobia

Food neophobia was assessed using the scale created by Pliner and Hobden [66] and its German translation by Siegrist et al. [91]. As per Lammers et al. [74], the ten items were scored on a 5-point Likert scale instead of the original 7-point Likert scale. Answer options ranged from ‘1 = I do not agree’ to ‘5 = I fully agree’ and displayed high internal consistency (α = 0.79). An example of the included items is ‘I don’t trust new foods.’ [66].

##### Food Technology Neophobia

Based on Verbeke [72], food technology neophobia was assessed using four items from the original 13-item scale by Cox and Evans [67]. The German version of the scale was adopted from Lammers et al. [74]. The answer format was also modified following Lammers et al. [74] and Verbeke [72], so that respondents indicated their agreement with items such as ‘New food technologies decrease the natural quality of food.’ [67] on a 5-point Likert scale. The answer options ranged from ‘1 = I do not agree’ to ‘5 = I fully agree’. With a Cronbach’s α value of 0.82, the scale had high internal consistency.

##### Food Disgust

Food disgust was measured with the short version of the food disgust scale developed by Hartmann and Siegrist [64]. In line with studies of Lammers et al. [74] and Dupont and Fiebelkorn [50], we used the German 5-point Likert scale version ranging from ‘1 = Not disgusting at all’ to ‘5 = Totally disgusting’ with the scale introduced by the following phrase: ‘Please indicate how disgusting you find the following situations or products.’ The scale consisted of eight items, each representing a dimension of food disgust, such as (1) animal flesh, (2) poor hygiene, (3) human contamination, (4) mould, (5) decaying fruits, (6) fish, (7) decaying vegetables, and (8) living contaminants. The scale included statements such as ‘To put animal cartilage into my mouth.’ [64]. The food disgust scale had high internal consistency (α = 0.73).

##### Sensation Seeking

Sensation seeking was assessed by using the brief sensation seeking scale based on Hoyle et al. [92], which originates from the Sensation-Seeking Scale Form V [73]. The short version consists of the following four subscales: (1) thrill and adventure seeking, (2) experience seeking, (3) disinhibition, and (4) boredom susceptibility [91]. Two items for each subscale, thus a total of eight items, were used to measure sensation seeking. We adopted the German version from Lammers et al. [74]. The items were scored as in the original on a 5-point Likert scale ranging from ‘1 = I do not agree’ to ‘5 = I fully agree’ and included statements such as ‘I would like to explore strange places.’ [92]. The scale displayed high internal consistency (α = 0.81).

##### Green Consumption Values

The variable was measured using six items from the GREEN scale developed by Haws et al. [93]. The German version and the response format were adopted from Kusch and Fiebelkorn [76]. The items, such as ‘I am concerned about wasting the resources of our planet.’ [93], were answered on a 5-point Likert scale with answer options varying from ‘1 = I do not agree’ to ‘5 = I fully agree’. The scale had high internal consistency (α = 0.93).

### 3.3. Statistical Analysis

As a first step, we computed the mean (M) and standard deviation (SD) for all variables to obtain the first indications of their tendencies (Table 1). To analyse the normal distribution of all variables, their respective histograms and Q-Q plots were inspected. Furthermore, skewness and kurtosis were calculated (Table 1). All variables were distributed almost normally. In addition, the multivariate normal distribution was analysed. For this purpose, we used Mardia’s test, and a chi-square Q-Q plot was generated with the results showing no multivariate normal distribution.

To compare the mean values of the three behavioural intentions, namely willingness to (1) try, (2) buy, and (3) substitute, a *t*-test for connected samples was used. We performed a mediation analysis to assess whether general attitudes towards cultured meat influence willingness to consume a cultured meat burger and whether this influence is mediated by specific attitudes towards a cultured meat burger. To calculate the confidence intervals and inferential statistics, bootstrapping with 5000 samples along with heteroskedasticity-consistent standard errors was used. Based on the TPB, the analysis of a path model serves to identify significant predictors of willingness to consume as well as to account for supposed correlations between constructs of the TPB, based on the hypotheses (see Section 2 ‘Theoretical Framework: An Extended TPB’). In addition, a path analysis allows for a comparison of the various impacts of the independent variables [94]. Accordingly, the direct influences (described by standardized regression coefficients [94]) of the independent variables on the dependent variables were compared to rank the strength of influence.

Variables used in the path model were checked for multicollinearity by analysing their intercorrelations and calculating the variance inflation factor (VIF) and the tolerance. No correlation coefficients were above the critical value of 0.8, no VIF was greater than 10 and no tolerance value was below 0.2 (Table 1) [95]. Therefore, it can be assumed that no multicollinearity exists between the independent variables. The other requirements for calculating a path model such as independence, homoscedasticity, and normal distribution of the residuals [95] were also checked and could be confirmed. However, since the prerequisite of multivariate normal distribution was not given, the estimation method maximum likelihood with robust standard errors (MLR) was used to calculate the path model. The MLR is robust to the violation of the normal distribution and the independence of observations [96,97]. Although there is an assumed covariance between the added independent variables, it is neither displayed in nor will it be tested separately, since the lavaan package of the software R-Studio automatically assesses such correlations [98].

Statistical analyses were performed with IBM^®^ SPSS^®^ (Version 26) and the mediation analysis was computed with the extension *PROCESS* (Version 3.5.3) for IBM^®^ SPSS^®^ [99]. The path model was calculated with R-Studio; R package: lavaan (Version 1.2.1335).

## 4. Results

Of the respondents, 32.2% stated that they had already heard of cultured meat and that they knew what it meant. In contrast, 37.6% had heard of cultured meat but did not know what it meant. Accordingly, 30.2% of the participants had not yet heard of cultured meat. Respondents most frequently reported television (53.8%) as a source of information, followed by the internet (51.2%) and newspapers or journals (20.2%; Table 5). In total, 58.4% of the participants reported being willing to consume the cultured meat burger (willingness to consume > 4), of whom 50.3% were male and 49.7% female. As shown in Table 1, out of the nutritional-psychological variables, which were all measured on a 5-point Likert scale, sensation seeking (M = 2.40; SD = 0.75) and food neophobia (M = 2.54; SD = 0.60) showed a slightly negative mean compared to the scale centre. The respondents revealed moderate food technology neophobia (M = 3.19; SD = 0.84) and food disgust (M = 3.33; SD = 0.63). Furthermore, the respondents showed slightly positive green consumption values (M = 3.54; SD = 0.88) compared with the midpoint of the scale. In addition, the participants showed slightly positive general attitudes towards cultured meat (M = 3.44; SD = 0.70), as well as a slightly positive specific attitudes towards a cultured meat burger (M = 4.63; SD = 1.20), since these variables were measured on 5-point and 7-point Likert scales, respectively. Moreover, the respondents showed moderate perceived behavioural control (M = 4.15; SD = 1.96) and slightly positive willingness to consume a cultured meat burger (M = 4.34; SD = 1.92). Subjective norms had a nearly neutral mean (M = 4.12; SD = 1.63) compared to the scale midpoint.

### 4.1. Attitudes

A depiction of mean values on a single-item level allowed us to visualise the general attitudes towards cultured meat and the specific attitudes towards a cultured meat burger in more detail. Regarding general attitudes towards cultured meat, six of eight items measured at the scale midpoint were rated positively. The mean values of the two items ‘Cultured meat is highly nutritious.’ (M = 2.99; SD = 0.83) and ‘It is not natural for humans to eat cultured meat.’ (M = 2.68; SD = 1.17) were below the scale midpoint, with the latter having the most negative mean. In contrast, the item ‘No animals have to suffer when cultured meat is produced.’ had the most positive mean (M = 4.03; SD = 0.90).

Regarding specific attitudes towards a cultured meat burger, seven out of eight items were rated positively, as compared to the midpoint of the scale (see Figure 2 and Table 5). The adjective pair ‘Unhygienic—Hygienic’ had the highest mean (M = 5.22; SD = 1.35). Only the item consisting of the adjective pair ‘Artificial—Natural’ had a mean below the midpoint of the scale (M = 2.77; SD = 1.71; see also Figure 2 and Table 5).

### 4.2. Mediation Analysis

**Figure 3:**
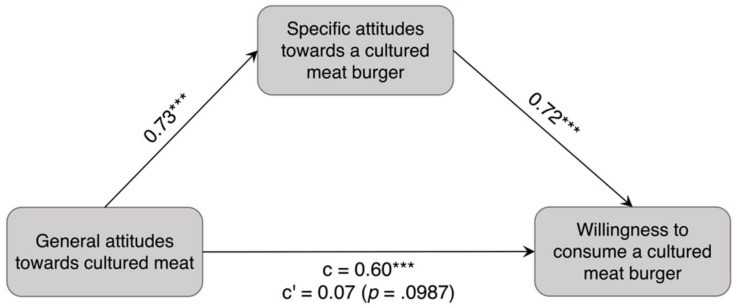
Model of mediation of the influence of general attitude towards cultured meat on the willingness to consume a cultured meat burger by specific attitude with a significant indirect effect: *ß* = 0.53, 95% BCa CI [0.46,0.60]. *** *p* < 0.001. Note: c = total effect of general attitudes towards cultured meat on willingness to consume without specific attitudes towards a cultured meat burger; c′ = direct effect of general attitudes towards cultured meat on willingness to consume with specific attitudes towards a cultured meat burger in the model.

In the mediation analysis (Figure 3), a significant total effect of general attitudes towards cultured meat on willingness to consume a cultured meat burger could be demonstrated (*ß* = 0.60, *p* < 0.001; Figure 3, c). After including the mediator specific attitudes towards a cultured meat burger in the model, general attitudes towards cultured meat were shown to significantly predict specific attitudes towards a cultured meat burger (*ß* = 0.73, *p* < 0.001), which in turn significantly predicted willingness to consume a cultured meat burger (*ß* = 0.72, *p* < 0.001). Furthermore, the mediation analysis revealed that after the inclusion of specific attitudes towards a cultured meat burger, general attitudes towards cultured meat no longer had a direct effect on willingness to consume a cultured meat burger (*ß* = 0.07, *p* = 0.100; Figure 3, c′). These findings means that the relationship between general attitudes towards cultured meat and willingness to consume a cultured meat burger is fully mediated by specific attitudes towards a cultured meat burger, indicating that there is a significant indirect effect (*ß* = 0.53, 95% bias-corrected and accelerated (BCa) CI [0.46, 0.60]).

### 4.3. Path Model

**Figure 4:**
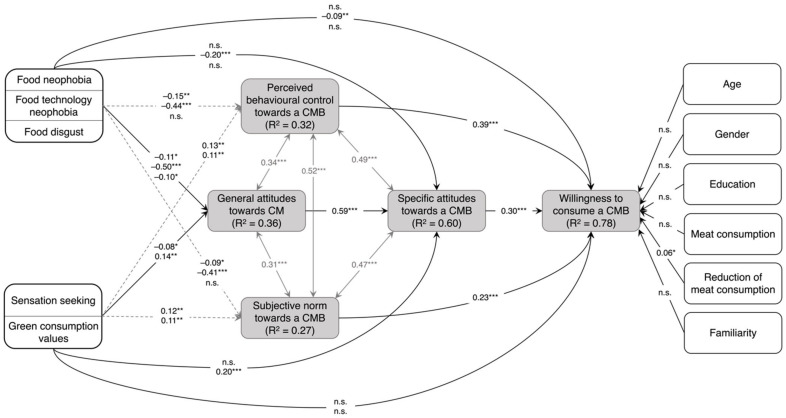
Path diagram examining interrelations between the TPB constructs (grey) and extended variables (white) and their influence on the willingness to consume a cultured meat burger. Listed values appear in order of the listed variables. Directional arrows represent relationships, and grey double arrows show correlations between the variables. Fit: CFI = 0.98, RMSEA = 0.06, SRMR = 0.03. * *p* < 0.05; ** *p* < 0.01; *** *p* < 0.001; n.s. = Not Significant. Note: CM = Cultured meat, CMB = Cultured meat burger, R^2^ = explained variance.

Figure 4 displays a path diagram based on a path model with standardised path coefficients. The following fit indices were used to evaluate the model fit: Comparative Fit Index (CFI), Root Mean Square Error of Approximation (RMSEA), and Standardised Root Mean Square Residual (SRMR) [100]. The fit indices suggest a good global fit: CFI = 0.98, RMSEA = 0.06, and SRMR = 0.03 [100,101]. The overall model explained 77.8% of the variance of willingness to consume a cultured meat burger. In total, 35.6% and 60.0% of the variance in general attitudes towards cultured meat and specific attitudes towards a cultured meat burger, respectively, could be explained. Moreover, the model explained 26.6% and 32.4% of the variance for subjective norms and perceived behavioural control, respectively.

Perceived behavioural control (*ß* = 0.39; *p* < 0.001) had the strongest influence on willingness to consume a cultured meat burger, followed by specific attitudes towards a cultured meat burger (*ß* = 0.30; *p* < 0.001) and subjective norms (*ß* = 0.23; *p* < 0.001). Out of the nutritional-psychological variables, only food technology neophobia (*ß* = –0.09; *p* < 0.010) significantly influenced willingness to consume. In addition, among the sociodemographic variables, only the intention to reduce meat consumption had a negative influence on the willingness to consume a cultured meat burger (*ß* = –0.07; *p* < 0.05). Considering general attitudes as dependent variable, all nutritional-psychological variables significantly affected general attitudes towards cultured meat, with food technology neophobia displaying the greatest negative influence (*ß* = –0.50; *p* < 0.001) followed by food neophobia (*ß* = –0.11; *p* < 0.05) and food disgust (*ß* = –0.10; *p* < 0.05), as well as sensation seeking (*ß* = –0.08; *p* < 0.05). The only positive predictor of general attitudes towards cultured meat was green consumption values (*ß* = 0.14; *p* < 0.01). Moreover, general attitudes towards cultured meat (*ß* = 0.59; *p* < 0.001) and green consumption values (*ß* = 0.20; *p* < 0.001) were positive predictors of specific attitudes towards a cultured meat burger. In contrast, food technology neophobia was the only negative predictor for specific attitude towards a cultured meat burger *(ß* = –0.20; *p* < 0.001).

## 5. Discussion

This study found that 58.4% of German participants reported being willing to consume a cultured meat burger. In total, 65% of the respondents reported being willing to try a cultured meat burger, with 49.5% being willing to buy it and 46.7% being willing to substitute conventional meat with a cultured meat burger. Similar results for willingness to buy cultured meat were found in a paper by Weinrich et al. [61] with German adults, as well as willingness to consume a cultured meat burger in a study by Dupont and Fiebelkorn [49] with German children and adolescents. However, the findings of this study show a greater reported willingness to buy a cultured meat burger in comparison with a previous German survey, which found that only 17% of German respondents were willing to buy cultured meat if it was available in supermarkets [102]. The much lower values of the Forsa [102] survey were probably caused by the wording of the item in the study. First, the description of cultured meat as meat on a test tube may have had a deterrent effect [69], and second, only the substitution of meat was queried in the questionnaire, which is much more difficult to implement in everyday life than, for example, just trying cultured meat. The difference in the willingness to buy could also be because the study of Forsa [102] is older than the present study and the study by Weinrich et al. [61]. However, this assumption would need to be checked by longitudinal studies. Another reason for the difference could be that this study used a bipolar scale, which can be associated with a tendency toward extreme response options [103], to survey the willingness to buy a cultured meat burger, whereas the Forsa study used a nominal scale.

### 5.1. Aptitude of the Extended Theory of Planned Behaviour

The path model confirmed the usefulness of the extended TPB and its added variables to predict willingness to consume a cultured meat burger of German participants. The TPB alone accounted for 76.6% of the variance in willingness to consume a cultured meat burger, while the model overall explained 77.8% of the variance. A study by Onwezen et al. [27] showed similar findings for TPB concerning the acceptance of burgers made from cultured meat.

In this study, specific attitudes towards a cultured meat burger not only showed a direct positive influence on willingness to consume a cultured meat burger but also fully mediated the influence of general attitudes towards cultured meat on willingness to consume a cultured meat burger. Specific attitudes towards a cultured meat burger and general attitudes towards cultured meat were collected at two different measurement levels in this study. Previous studies have shown that the level of measurement is crucial for the predictive power of the factors [104]. In addition, the compatibility of the two variables with the willingness to consume a cultured meat burger has also differed due to the specificity of the target of the two attitudes [51,87]. General attitudes targeted cultured meat without a specific product category, while specific attitudes—as well as perceived behavioural control, subjective norms, and the willingness to consume—had a specific product as the target, namely a cultured meat burger. Thus, the difference in the specificity of the attitude variables in relation to the willingness to consume a cultured meat burger could explain why general attitudes towards cultured meat have only an indirect influence on the willingness to consume the burger [51]. In addition, compared to the general attitudes towards cultured meat, the specific attitudes towards a cultured meat burger represent a more specific evaluation. This narrower view could also be a reason why the impact of general attitudes towards cultured meat is moderated by the more specific attitudes towards a cultured meat burger. Contrary to our assumption, specific attitudes towards a cultured meat burger did not have the strongest influence on willingness to consume a cultured meat burger.

Regarding both attitude scales on a single-item level, the respondents perceived cultured meat or the cultured meat burger as artificial, as opposed to natural. Thus, the perceived unnaturalness associated with cultured meat was the greatest concern identified in this study. According to Laestadius [105], the perceived unnaturalness of cultured meat may be linked to the evaluation of cultured meat and the technologies for its production as unethical. This claim is supported by the negative influence of food technology neophobia on general attitudes towards cultured meat and specific attitudes towards and willingness to consume a cultured meat burger (Figure 4). Anchoring cultured meat to more favourable associations by using product names that sound less technological, such as ‘clean meat’ or ‘animal-free meat’ [105,106], leads to more positive attitudes towards cultured meat [14]. Moreover, the media coverage focusing on environmental and sustainability aspects of the production of cultured meat [63] may increase willingness to consume a cultured meat burger for German consumers.

This study there revealed a positive effect of subjective norms on willingness to consume a cultured meat burger. However, subjective norms showed the weakest influence compared to perceived behavioural control and specific attitudes towards a cultured meat burger on willingness to consume a cultured meat burger. The results support previous research that subjective norms are a weak predictor of the intention to consume organic [33] and sustainable foodstuff [46,107]. La Barbera and Ajzen [55] were able to show that a higher perceived behavioural control can reduce the influence of subjective norms on intention, while the influence of attitudes can be increased. Since the perceived behavioural control of the participants in this study was relatively high when compared to the scale midpoint, this could be a possible explanation for the low influence of subjective norms compared with perceived behavioural control and specific attitudes towards a cultured meat burger in this study. In addition, McEachan et al. [108] were able to show that the influence of subjective norms on dietary intention is higher in adolescents, while attitudes predict dietary intention more accurately in adults. In line with Vermeir and Verbeke [107], it, therefore, seems that the impact of subjective norms varies according to the personal values and characteristics of the individual.

Perceived behavioural control was the strongest predictor of willingness to consume a cultured meat burger, contradicting prior studies [27,46,107]. The strong influence of perceived behavioural control on willingness to consume a cultured meat burger may be attributable to respondents’ perceptions that adding a cultured meat burger to their dietary habits would be difficult. In the questionnaire, we specifically asked participants how easy it would be to integrate a cultured meat burger into their diet. The respondents may have perceived that the availability of a cultured meat burger is limited because its large-scale production is still developing, and it is not commercially available yet [16]. The construct specifically examined the integration of cultured meat for a cultured meat burger, so the food choice was limited to just one product. Hence, participants with an aversion to burgers, regardless of their meat content, were restricted in their choice. In addition, in previous studies, respondents have shown scepticism about the feasibility of cultured meat [56,79,109]. Thus, the prospective cultured meat products must be both readily available and affordable for consumers [107]. Although the production cost of the first cultured beef burger patty was $325,000 in 2013 [15], it now seems possible to offer prices to consumers that match those of high-end restaurants. For example, Eat Just’s cultured chicken nuggets were priced at $23 in the restaurant 1880 in Singapore in December 2020 [110]. Hence, conditions must be created that facilitate the access to and purchase of cultured meat [35] to raise the perception of its availability. Fulfilling these conditions with the appearance of cultured meat on the market may lead to increased perceived behavioural control and willingness to consume, respectively.

### 5.2. Influence of the Extended Variables

#### 5.2.1. Influence of Sociodemographic Variables and Eating Habits

Contrary to our assumptions, none of the constructs from the sociodemographic variables, had a significant impact on the willingness to consume a cultured meat burger. As seen in a study by Weinrich et al. [61] with German participants, sociodemographic data on age and gender did not show any influence on the acceptance of cultured meat. In addition, the results are consistent with findings indicating no significant influence of age on the acceptance of cultured meat [53,62]. The impact of gender in this study could also be masked by the somewhat higher education level of the respondents. Hence, in research by Valente et al. [111] with a sample that had a high level of education, no difference in gender could be shown in the willingness to eat cultured meat. Accordingly, it may be that gender does not play a role in the acceptance of a cultured meat burger among respondents with a high level of education. Moreover, in a paper by Slade [59], gender showed an influence on the willingness to purchase a cultured meat burger only in a model with sociodemographic determinants, but not in the overall model (including variables on sociodemographics, shopping behaviour, attitudes towards technologies, and general attitudes). This may indicate that the gender effect might be masked by the effect of other variables in the model. Furthermore, as per Weinrich et al. [61], the educational level of the German participants was higher in this study compared to the general German population. Weinrich et al. [61] were also unable to demonstrate a direct influence of the educational level on the acceptance of cultured meat. However, the level of education showed an influence on the prior knowledge of respondents, which in turn positively influenced attitudes towards the ethical benefits of cultured meat [61]. These findings could also explain the lack of direct influence of educational level on willingness to consume a cultured meat burger.

In this study, no influence of meat consumption on willingness to consume a cultured meat burger could be demonstrated. Thus, the results of previous studies could not be confirmed [50,53]. On the one hand, meat consumption might not have any influence on the willingness to consume for cultural reasons. For example, Bryant et al. [53] were able to demonstrate an influence of meat consumption frequency for Indian and Chinese respondents, but not for American participants. In addition, Dupont and Fiebelkorn [50] surveyed German children and adolescents, which suggests that the influence of meat consumption could also be dependent on the age of the participants.

The intention to reduce one’s consumption of meat proved to be the only predictor with a negative influence on willingness to consume a cultured meat burger among the variables examining eating habits. Participants who were less willing to reduce their meat consumption showed a higher willingness to consume a cultured meat burger. This observation corresponds to previous findings in surveys of Asian respondents: Compared to vegetarians, consumers of conventional meat showed a higher willingness to purchase cultured meat [53]. Moreover, findings by Vanhonacker et al. [112] suggest that consumers who intend to reduce their meat consumption generally prefer to forgo meat instead of considering a substitute meat product. Since cultured meat, based on its definition, is closely related to conventional meat [113], it may not be an acceptable substitute for conventional meat for people who want to reduce their meat consumption.

#### 5.2.2. Influence of Nutritional-Psychological Variables

Contrary to expectations, familiarity with cultured meat did not prove to affect willingness to consume a cultured meat burger. With our methodology, we could only clarify whether the respondents already knew about the existence of cultured meat. We could not clarify to what extent they had in-depth knowledge about the technology behind the production and the potential advantages and disadvantages of eating cultured meat in terms of sustainability. Thus, a more differentiated survey of familiarity with cultured meat, such as in Bryant et al. [53], might provide a deeper insight into the mechanisms by which familiarity affects the willingness to consume a cultured meat burger. The prior knowledge of the participants about cultured meat was not further differentiated in the questionnaire, as it only surveyed whether the participants had already heard of cultured meat. This could indicate that only the familiarity with the concept of cultured meat has no influence, but possibly deeper knowledge about the production and the advantages and disadvantages could show an influence.

In line with our hypothesis, food disgust proved to be a negative predictor for general attitudes towards cultured meat. According to previous findings, food disgust is not only caused by the expected sensory properties of cultured meat [23,78,79] but may also be linked to novel food technologies and their perceived unnaturalness [70,78,79]. In consequence, to lessen the disgust reaction, descriptions of cultured meat should be less technical [70]. Moreover, the focus should not be on the differences between cultured and conventional meat, but on the similarities between the two [69,114]. Another helpful marketing approach could be to emphasise that the production of cultured meat is no more unnatural or disgusting than that of conventional meat [105], given the conditions in modern animal husbandry that have little to do with the natural living conditions of pigs [15].

Food neophobia only had a negative influence on general attitudes towards cultured meat, but not on the willingness to consume a cultured meat burger. However, as general attitudes towards cultured meat were shown to have a positive indirect effect on the willingness to consume a cultured meat burger in this study, food neophobia should be reduced to improve attitudes and consequently increase the willingness to consume cultured meat burgers. Therefore, particularly in childhood and adolescence, opportunities should be provided to learn about and try new foods to reduce food neophobia [115]. In addition, sensory training [116] and taste training [117] could be useful approaches to reduce food neophobia.

Food technology neophobia was the most significant negative predictor of both general attitudes towards cultured meat and specific attitudes towards a cultured meat burger. Moreover, food technology neophobia was a direct negative predictor of willingness to consume a cultured meat burger. This result corresponds to previous findings indicating that unnaturalness related to cultured meat is a central issue for consumers [68,70,118]. The technology used to produce cultured meat raises questions about its ethical justifiability as well as concerns about personal health [105]. According to Marcu et al. [118], the processes of this new technology must be made more transparent and understandable for laypersons This initiative could lead to more transparency regarding the production process [69] and might have a positive impact on attitudes towards cultured meat and willingness to consume a cultured meat burger.

Contrary to our hypothesis, sensation seeking turned out to be a negative predictor of general attitudes towards cultured meat but showed no influence on willingness to consume a cultured meat burger. At first glance, the results seem counterintuitive, as cultured meat is a novel and interesting alternative to meat. However, respondents who were less willing to experience new adventures had a more positive attitude to cultured meat. We presume that the respondents might have perceived cultured meat as less exciting than, for example, insect-based foods, as it resembles conventional meat in its appearance and at least partly falls within its definition [113].

As expected, green consumption values were a positive predictor for general attitudes towards cultured meat and specific attitudes towards a cultured meat burger but showed no influence on willingness to consume a cultured meat burger. This result concurs with previous findings that people’s attitudes towards cultured meat are positively influenced by the perception that cultured meat is a sustainable alternative to conventional meat [62,78,79,80,119]. Moreover, respondents’ perceptions that cultured meat decreases resource use [79], such as land or water use [24], and its perceived positive impact on climate change positively affect their attitudes [79]. This finding is also underlined by the fact that the items of the scale on specific attitudes towards a cultured meat burger that relate to sustainability and environmental aspects of a cultured meat burger were rated particularly positively. Therefore, marketing strategies that highlight the environmental and sustainability benefits of a cultured meat burger might change attitudes towards and promote the acceptance of the product.

### 5.3. Limitations of the Study

This study had several limitations. First, its limited representativeness should be mentioned, as the sample’s mean age and educational standard are slightly higher than that of the German population.

Moreover, due to self-selection, participants within a panel could choose to answer particular surveys [120]. Thus, responses were possibly obtained from participants who already showed some interest in the topic of cultured meat.

Note that this study measured the intention behind the consumption of cultured meat and not the behaviour itself. Hence, our results do not capture the actual execution of a behaviour. According to Vermeir and Verbeke [121], situational and product-related aspects, among others, can strongly influence or even hinder the consumption of a product, even if there is a high willingness to consume.

Additionally, a statistical limitation is that perceived behavioural control was measured by one item and could be higher if measured with more items.

Furthermore, all constructs of the TPB in this study, except general attitudes towards cultured meat, refer to the specific product of a cultured meat burger; therefore, the results are also limited to the consumption of a cultured meat burger. The findings of this study are not necessarily transferrable to the willingness to consume cultured meat in general because the nature of future cultured meat products may vary [15]. For example, Wilks and Phillips [62] have already demonstrated that respondents were more likely to consume cultured beef than other cultured products such as cultured fish or cultured pork. Consequently, future studies in Germany should investigate the acceptance of other cultured meat products, such as cultured chicken nuggets to gain a more comprehensive understanding of the factors influencing cultured meat products in general. Furthermore, it would also be useful to conduct longitudinal studies on the acceptance of cultured meat in Germany to identify emerging or declining trends in the acceptance of cultured meat at an early stage and to be able to interpret them better.

Another limitation of the study is that the respondents were given a brief description of cultured meat at the beginning of the questionnaire. Furthermore, in the heading of the questionnaire, the term laboratory meat was used. It has already been shown by Bryant and Dillard [69] that different framings of cultured meat have different influences on the willingness to consume it and on attitudes towards the product. Therefore, the description’s influence on the respondent cannot be excluded, although the text was formulated as neutrally or as positively as possible.

## 6. Conclusions

This study demonstrates that the TPB is an adequate model to predict and explain the main determinants of the willingness to consume a cultured meat burger in Germany. Moreover, by expanding the TPB with other variables, more variance in acceptance can be explained. Furthermore, it could be confirmed that background factors in the TPB are primarily mediated by attitudes. Although the examined nutritional-psychological variables were able to explain the additional variance, the effects were relatively small.

As perceived behavioural control had the strongest impact on willingness to consume a cultured meat burger, it is essential that cultured meat products be both readily available and affordable to consumers [107]. In addition, the accessibility and purchasing conditions [35] of cultured meat products should be facilitated to broaden the perception of availability, thus increasing perceived behavioural control and willingness to consume cultured meat products. As specific attitudes towards the cultured meat burger also had a strong influence on willingness to consume, improving attitudes could lead to an increased willingness to consume cultured meat burgers [31,46,79,107]. In this respect, media coverage focusing on the environmental and sustainability aspects of cultured meat production [63] might improve attitudes as well as willingness to consume cultured meat products among German consumers. Moreover, media coverage should not be focused on differences between cultured meat burgers and conventional meat burgers; instead, similarities should be highlighted [69,114].

As a high degree of unnaturalness was associated with cultured meat or a cultured meat burger in this study, communications and marketing of cultured meat products should therefore provide a more natural and positive image of cultured meat. Concerns about the unnaturalness of cultured meat should be addressed to encourage consumers to familiarise themselves with the product and change their perceptions towards it. One way this could be achieved is by using terminology and product labelling that is less technological [69]. Furthermore, there should be transparency about the production of cultured meat, and a dialogue should be created about the fears associated with the new technology and the benefits that arise from it. Addressing consumers’ critical questions in marketing strategies or information campaigns could lead to a higher public understanding of the technology behind cultured meat production [118], which in turn could lead to higher acceptance. Overall, the above aspects show that the barriers to the acceptance of cultured meat products cannot be overcome by removing a single obstacle, but by creating an open dialogue about the fears and hopes involved in this new sustainable alternative to conventional meat.

## Figures and Tables

**Table 1:** Spearman’s correlation coefficients and descriptive statistics of the independent and dependent variables (*n* = 497).

Variable	1	2	3	4	5	6	7	8	9	10	11	12^1^	13^1^	14^1^	15^1^
(1) Age	1	−0.27 ***	−0.04	0.09	−0.08	0.17 ***	0.14 **	0.03	−0.29 ***	0.01	0.07	0.01	−0.05	−0.16 ***	−0.09 *
(2) EDU		1	−0.01	−0.02	0.24 ***	−0.14 **	−0.09 *	−0.11 *	0.14 **	0.12 **	0.07	−0.01	−0.01	0.03	−0.00
(3) MCON			1	0.10 *	0.04	−0.07	0.01	−0.15 ***	0.03	−0.31 ***	0.03	−0.06	0.06	0.00	0.04
(4) MRED				1	−0.12 **	0.10 *	0.12 **	−0.04	−0.18 ***	−0.30 ***	−0.09	−0.23 ***	−0.17 ***	−0.23 ***	−0.22 ***
(5) FAM					1	−0.12 **	−0.13 **	−0.18 ***	0.11 *	0.18 ***	0.25 ***	0.21 ***	0.12 **	0.12 **	0.13 **
(6) FN						1	0.31 ***	0.36 ***	−0.29 ***	−0.15 **	−0.30 ***	−0.28 ***	−0.28 ***	−0.33 ***	−0.35 ***
(7) FTN							1	0.12 **	−0.10 *	−0.10 *	−0.58 ***	−0.55 ***	−0.45 ***	−0.51 ***	−0.55 ***
(8) FD								1	−0.18 ***	0.00	−0.18 ***	−0.09	−0.08	−0.09 *	−0.08
(9) BSS									1	0.18 ***	0.02	0.11 *	0.19 ***	0.22 ***	0.19 ***
(10) GCV										1	0.17 ***	0.34 ***	0.21 ***	0.18 ***	0.24 ***
(11) ATT											1	0.71 ***	0.50 ***	0.53 ***	0.62 ***
(12) SATT												1	0.66 ***	0.71 ***	0.79 ***
(13) SN													1	0.65 ***	0.73 ***
(14) PBC														1	0.80 ***
(15) WTC															1
Items	1	1	1	1	1	10	4	8	8	6	8	8	3	1	3
α						0.79	0.82	0.73	0.81	0.93	0.85	0.91	0.93		0.94
M	49.93	4.87	3.71	1.47	2.02	2.54	3.19	3.33	2.40	3.54	3.44	4.63	4.12	4.15	4.34
SD	16.23	1.15	1.16	0.63	0.79	0.60	0.84	0.63	0.75	0.88	0.75	1.20	1.63	1.96	1.92
Skewness	−0.04	−0.37	−0.08	0.99	−0.04	0.33	−0.12	0.11	0.40	−0.53	−0.19	−0.29	−0.31	−0.23	−0.41
Kurtosis	−1.08	−1.35	0.27	−0.10	−1.40	0.45	−0.00	−0.23	−0.11	0.20	0.02	0.07	−0.65	−1.07	−1.02

Note: * *p* < 0.05, ** *p* < 0.01, *** *p* < 0.001. EDU = Education, MCON = Meat consumption, FAM = Familiarity, FN = Food neophobia, FTN = Food technology neophobia, FD = Food disgust, BSS = Sensation seeking, GCV = Green consumption values, ATT = General attitudes towards cultured meat, SATT = Specific attitudes towards a cultured meat burger, SN = Subjective norms towards a cultured meat burger, PBC = perceived behavioural control towards a cultured meat burger, WTC = Willingness to consume a cultured meat burger, M = Mean, SD = Standard deviation, α = Cronbach’s alpha. ^1^ Scale recoded for evaluation: –3 = 1; +3 = 7.

**Table 2:** Overview and description of the willingness to consume a cultured meat burger (*n* = 497).

Variable ^1^	Response Format ^2,3^	Mean Value (*SD*)
Willingness to try a cultured meat burger (WTT)	7-point bipolar scale (−3 = very unlikely; +3 = very likely)	4.88 (2.03)
Willingness to buy a cultured meat burger (WTB)	7-point bipolar scale (−3 = very unlikely; +3 = very likely)	4.06 (2.05)
Willingness to use a cultured meat burger as a substitute (WTS)	7-point bipolar scale (−3 = very unlikely; +3 = very likely)	4.08 (2.02)
Willingness to consume a cultured meat burger		4.34 (1.92)

Note: The variable ‘willingness to consume a cultured meat burger’ has been calculated as an aggregated mean based on the mean values of the variables ‘willingness to try’, ‘willingness to buy’ and ‘willingness to substitute’. ^1^ Source of the variables: Lammers et al. [74]. ^2^ Results of the *t*-test for connected samples: WTB—WTT: *T* = 16.18, *p* < 0.001; WTS—WTT: *T* = −13.07, *p* < 0.001; WTS—WTB: *T* = −0.40, *p* = 0.690. ^3^ Scale recoded for evaluation: −3 = 1; +3 = 7.

**Table 3:** Single items of the general attitudes towards cultured meat (*n* = 497).

Item	Mean (*SD*)
No animals have to suffer when cultured meat is produced.	4.03 (0.90)
Cultured meat carries harmful microbes. ^R^	3.73 (0.92)
It is unconscionable to produce cultured meat. ^R^	3.63 (1.08)
Cultured meat contains harmful toxins. ^R^	3.56 (0.94)
Eating cultured meat will increase the risk of infectious disease. ^R^	3.54 (1.00)
Eating cultured meat is disgusting. ^R^	3.39 (1.09)
Cultured meat is highly nutritious.	2.99 (0.83)
It is not natural for humans to eat cultured meat. ^R^	2.68 (1.17)

Note: The items were scored on a 5-point Likert scale. Answer options ranged from ‘1 = I do not agree’ to ‘5 = I fully agree’. R marks reverse-coded items.

**Table 4:** Single items of the specific attitudes towards a cultured meat burger (*n* = 497).

Adjective Pairs	Mean (*SD*) ^1^
unhygienic—hygienic	5.22 (1.35)
environmentally harmful—environmentally helpful	5.15 (1.44)
unsustainable—sustainable	5.15 (1.51)
dirty—clean	5.14 (1.37)
not interesting—interesting	4.86 (1.87)
disgusting—delicious	4.43 (1.56)
unhealthy—healthy	4.32 (1.47)

Note: The items were rated on a 7-point bipolar scale, with the smallest value linked to a negative connotation (e.g., –3 = unhygienic) and the highest value to a positive connotation (e.g., +3 = hygienic). ^1^ Scale recoded for evaluation: –3 = 1; +3 = 7.

**Table 5:** Respondents´ sources of information about cultured meat (*n* = 347).

Categories	Frequency (Mentions)
Television	53.8% (184)
Internet	51.2% (175)
Newspaper/Trade magazine	20.2% (69)
Friends/Acquaintances	12.% (41)
Radio	0.3% (1)
Cinema	0.3% (1)

## Data Availability

The data presented in this study are available within the article.
